# Rethinking ‘Recovery’: A Comparative Qualitative Analysis of Experiences of Intensive Care With COVID and Long Covid in the United Kingdom

**DOI:** 10.1111/hex.70253

**Published:** 2025-04-09

**Authors:** Alice MacLean, Annelieke Driessen, Lisa Hinton, Sarah Nettleton, Cervantee Wild, Eilidh Anderson, Ashley Brown, Pat Hoddinott, Callum O'Dwyer, Sue Ziebland, Kate Hunt

**Affiliations:** ^1^ Institute for Social Marketing and Health University of Stirling Stirling UK; ^2^ Department of Health Services Research and Policy London School of Hygiene and Tropical Medicine London UK; ^3^ Nuffield Department of Primary Care Health Sciences University of Oxford Oxford UK; ^4^ Department of Anthology University of Amsterdam Amsterdam Netherlands; ^5^ THIS Institute University of Cambridge Cambridge UK; ^6^ Department of Sociology University of York York UK; ^7^ Department of Paediatrics: Child and Youth Health, School of Medicine University of Auckland Auckland New Zealand; ^8^ NMAHP Research Unit University of Stirling Stirling UK; ^9^ Lived experience coinvestigator, UK

**Keywords:** COVID19, illness narrative, intensive care, Long Covid, recovery

## Abstract

**Introduction:**

Interpretations of ‘recovery’ from illness are complex and influenced by many factors, not least patient expectations and experiences. This paper examines meanings of ‘recovery’, and how it is strived towards, drawing on the example of COVID‐19 infection.

**Methods:**

Drawing on qualitative interviews (*n* = 93) conducted in the UK between February 2021 and July 2022, we compare adults' accounts of being admitted to an Intensive Care Unit (ICU) with COVID‐19 to accounts of being ill with Long COVID, defined as ongoing symptoms for at least 12 weeks postinfection. We conducted a multi‐stage comparative analysis using Nvivo to organise and code the data.

**Results:**

We identified similarities and differences in participants' descriptions of their ‘worlds of illness’. For both groups, perceptions of recovery were shaped by the novel, unknown nature of COVID‐19. Participants questioned the achievability of full restoration of prior states of health, highlighted the heterogeneity of ‘recovery trajectories’ and described the hard physical and emotional work of adjusting to changed selves. Themes that revealed differences in ‘worlds of illness’ described included the different baselines, waymarkers, and pathways of illness experiences. Differences in other people's responses to their illness were also evident. For ICU participants, hospitalisation, and especially ICU admission, conferred legitimate patient status and authenticity to their symptoms. Family, friends and healthcare professionals acknowledged their illness, celebrated their survival, and granted them latitude to recover. For Long Covid participants, their patient status often lacked comparable authenticity in others' eyes. They reported encountering a lack of recognition and understanding of their ongoing need to recover.

**Conclusions:**

This study highlights how the meanings of illness ascribed by others can influence how recovery is experienced. Our findings highlight the importance of ensuring people are made to feel their illness experiences are legitimate, regardless of hospitalisation status, formal diagnosis or lack of medical knowledge and pathways. They also indicate the value of emphasising the different permutations, and lack of linearity, that recovery can take. This may help to help to guard against a lack of understanding for experiences of recovery which do not meet idealised notions.

**Patient or Public Contribution:**

Both studies were guided by an advisory panel that included patient and public involvement representatives with lived experience of Intensive Care/COVID experience and Long COVID respectively. Through regular meetings with the research teams, the advisory panel had input into all aspects of the study conduct, including recruitment methods and content of the interview topic guide and feedback on preliminary analyses. The Long COVID study also included a lived experience coinvestigator who contributed to data interpretation and analysis.

## 1 Introduction

If illness or injury cannot be avoided, recovery becomes an understandable ambition. In the early months of the COVID‐19 pandemic it was anticipated that ‘the great majority’ of people infected with COVID‐19 would experience ‘mild‐to‐moderate’ illness ‘similar to seasonal flu’, while a ‘minority [would] develop complications severe enough to require hospital care [which for] a small proportion […]may be severe enough to lead to death’ [1]. ^(p.5)^ At that time it was not realised that this mild vs severe classification was too simplistic and could not account for those who did not recover as expected and went on to experience long term illness [2, 3]. A ‘mild’ vs ‘severe’ dichotomy of illness presupposes particular needs and pathways of recovery, thereby limiting what stories can be told, and what experiences can be conveyed. In turn, this risks ‘epistemic injustice’ [4], where some accounts of ‘recovery’ are privileged while others are neglected or rejected [5, 6]. This article examines meanings of ‘recovery’ and how it is strived towards, using the example of COVID‐19 infection. We draw on interviews, conducted in the UK between February 2021 and July 2022, to compare accounts of striving towards ‘recovery’ by adults who were critically ill and admitted to an Intensive Care Unit (ICU) or had Long COVID.

### 1.1 COVID‐19, ICU and Long COVID

Internationally, the COVID‐19 pandemic gave rise to increased ICU admissions, and unprecedented numbers of critical care survivors [7, 8]. Survivors of critical illness can experience multidimensional disability and require rehabilitation following discharge from ICU [7]. Long COVID, an often‐contested condition [9] with 200 fluctuating symptoms spanning 10 organ systems [10], began to emerge during 2020 [3, 11, 12]. However, Long COVID displays significant heterogeneity in symptom experiences and illness trajectory, and hence there is no typical Long COVID patient [13]. To date, Long COVID occupies ‘a category of the medically unknown [and] a diagnosis of exclusion’. [14] ^(p.11)^ In March 2024, statistics reported that 2 million people were experiencing Long COVID in the UK; 71.1% had experienced symptoms for at least a year and 51.3% for at least 2 years [15].

### 1.2 Problematising ‘Recovery’

A single understanding of what recovery from illness means is lacking, and may even be undesirable. Interpretations of recovery are complex and influenced by many factors, not least patient expectations and experiences [16]. Yet some argue that certain narratives, such as ‘success stories’, are preferentially told, heard and valued, whereas narratives of ongoing, relapsing or episodic illness are more likely to go untold or afforded lesser validity [17, 18]. In the context of novel, contested, and/or invisible illnesses, it is especially hard to occupy the ‘sick role’ [19]. In relation to depression, O'Brien [20] ^(p.573)^ argues that recovery is positioned as ‘an exercise of individual responsibility to return to a functioning and productive norm and prevent recurrence’. She suggests a ‘recovery imperative’, which neglects individual biography, values, goals, and social positioning, underpins notions of expected recovery, and imbues slow recovery or relapse with a sense of failure. Thus the ‘recovery imperative’ gives rise to a harmful discourse, positioning those who struggle to recover as not ‘trying’ hard enough [21].

### 1.3 Healthcare and ‘Recovery’ in the Context of COVID‐19

Research on the healthcare experiences of those who were critically ill with COVID‐19 during 2020–22 highlights many positive aspects of ICU care despite pandemic disruption, including relationships of reciprocal care, admiration, and gratitude for healthcare professionals [22, 23, 24, 25]. However, the evidence around ICU discharge and transition to recuperating at home highlights a deterioration in guidance and continuity of care [7, 26]. Over the same period, people with Long COVID reported predominantly negative healthcare experiences, including a struggle for recognition of their illness [3], having their candidacy for care rejected or diverted, leading them to feeling disbelieved and even abandoned by medical professionals, perhaps particularly among ‘vanguard patients’ who developed Long COVID early in the pandemic [27].

While after the initial months of the pandemic, there were clearer healthcare pathways and an immediate sense of urgency to develop treatments for people admitted to ICU with COVID‐19 [28], validated treatment pathways or those with Long COVID have been slow to materialise [10]. Cheston et al. [11] attribute this partly to ‘a deprioritisation of the chronic when dealing with the ongoing life‐threatening emergency of the acute’. Many with Long COVID have reported managing their symptoms without guidance or support from healthcare professionals, resulting in them experimenting with pacing (i.e. doing tasks in repeated bursts, planning activities so that pauses can be built in, prioritising valued tasks over others) and other adjustments [29, 30]. However, pacing and resting are less accessible symptom management options for people with job inflexibility or financial insecurity [31], which impacts their recovery [32, 33].

In relation to recovery from COVID‐19 critical illness, Onrust et al. [25] who administered questionnaires to ICU survivors (*n* = 56) and family members (*n* = 67) and conducted interviews with survivors (*n* = 6), reported that participants perceived returning to paid employment as representing recovery and a return to ‘normality’. Gonçalves et al. [7] (whose 23 interviewees included health professionals, patients critically ill with COVID‐19 and relatives), argue that recovery implies being ‘cured’ which does not align with the concepts of ‘survivorship’ and ‘recalibration’ that participants described.

Summarising Long COVID studies with 2‐3 years quantitative follow‐up data, Al‐Aly & Topol [34] ^(p.831)^ state “spontaneous recovery or return to baseline status is uncommon”. Data from the UK‐based REACT study (*n* = 242,712) showed that only 31% of those with persistent symptoms at 12 weeks had recovered within a year [35]. ^(p.3)^ Qualitative explorations of Long COVID reveal complex patterns of ‘recovery’, such as varying understandings of what ‘getting back to normal’ entails [36] and how the ‘ongoing unknowns’ of the condition can impact the ability of people with Long COVID to (re)construct a coherent and stable sense of self [37]. ^(p.5)^ Qualitative studies and syntheses highlight evidence which disrupts any idea that recovery from Long COVID follows a linear transition between binary states of illness and wellness [38, 39].

Our aim here is to add to understandings of the ways in which recovery from illness is understood and strived for by comparing the accounts of participants who were admitted to ICU with critical COVID‐19 illness (ICU participants) with the accounts of participants who experienced Long COVID (LC participants).

## 2 Materials and Methods

### 2.1 Design and Theoretical Approach

The data are from in‐depth narrative interviews (*n* = 93) from linked qualitative UK studies on COVID‐19 which used identical methods of data collection and overlapping teams. The studies were conducted between February 2021 and July 2022. Ethical approval was granted by Berkshire Ethics Committee (12/SC/0495).

### 2.2 Participants

Sampling in the studies aimed to achieve maximum variation [40] by including participants of diverse ages, occupations, ethnicity, gender, and geographical locations. The researchers recruited from a variety of sources including clinicians, support groups and social media, and employed snowball sampling when accessing ‘hardly reached’ groups (e.g., ethnic minorities). For a discussion on the term ‘hardly reached’ instead of ‘hard‐to‐reach’ see Sokol et al. [41].

The analysis reported here includes 30 adults who had been admitted to ICU with COVID‐19, and 63 adults with Long COVID. At the time of the interviews none of the ICU participants identified as having Long COVID. Participants' ages ranged from 20 to 80 years, with the majority (*n* = 65) aged between 30 and 59 years. The sample comprised a majority of female (*n* = 56) and white participants (*n* = 68 white British or other white).

### 2.3 Interviews

One researcher interviewed all ICU participants and a team of ten conducted the Long COVID interviews; all are experienced qualitative researchers trained in the same protocols. All participants gave informed verbal consent to participate and have their interview recorded. All but two interviews were conducted remotely via videoconferencing platforms or telephone to comply with any public health restrictions in place, minimise infection risk and accommodate participants' preferences and IT literacy. Remote interviews enabled participants to cancel interviews when too fatigued or unwell and/or split the interview across various time points to reduce participant burden. On average, interviews lasted between 60 and 90 min (range 20–210 min).

Interviewers invited participants to recount how they first became ill with COVID‐19 and how this had affected their lives, and then prompted further discussion using a semi‐structured topic guide, which included questions about their experiences of treatment (including post‐discharge for ICU participants), current health, future expectations of ‘recovery’ and coping with the aftermath of their illness.

Interview audio‐recordings interviews were transcribed verbatim and checked for accuracy. Participants had the opportunity to read and redact their transcript before assigning copyright to use extracts from interview recordings and transcripts in written publications, broadcasts and on the public facing Health Experiences Insights (HEXI) platform.

### 2.4 Data Analysis

All direct identifiers were removed from transcripts before they were imported into NVivo and coded systematically using a coding framework, developed by each research team, which included anticipated and unexpected themes in the data [42]. We held cross‐study discussions which identified important thematic commonalities and disparities across the datasets, specifically related to experiences of illness and perspectives on ‘recovery’. One researcher (AM) then conducted a comparative analysis using a mind‐mapping ‘one sheet of paper’ (OSOP) technique [43]. The more refined themes generated using this technique were written up descriptively and separately for ICU and LC participants to aid comparison by viewing similarities and differences side by side. Further discussions among the authorship team, informed by these descriptive accounts of the data and existing literature, helped refine the line of argument to include understandings of participants' accounts as descriptions of their ‘worlds of illness’ [44, 45] and to pay close attention to how areas of convergence and divergence in these worlds can inform understanding of illness experiences and meanings. Interview extracts (IEs) are used to illustrate the key themes (longer extracts are presented in Tables 1, 2, 3, 4, 5). Pseudonyms (assigned by the researchers) are used throughout.

**Table 1:** Losses, scars and unknown futures after COVID‐19.

IE 1.	*[I was] previously active, playing rugby […] living a normal life, and now […] everything has to be planned[like] “Where am I going to park [at the supermarket]?” […] Previously I could just leave the house without a concern [it's] hard to adjust to*. (Elliott, LC)
IE 2.	*Everything is different. What I feel, […] what I think, how I behave […] I have to think before I do anything. Even watching TV. […] It's like I'm a different person*. (Nina, ICU)
IE 3.	*[T]here's no clear answers really to say, “Well, this is going to happen and then that's going to happen” [and] you're left worried, thinking, “Oh, is this normal to be feeling like this? [Is it] expected or has [COVID] damaged something?”* (Cathie, LC)

**Table 2:** Adjusting to changed selves: The hard work of striving for ‘recovery’.

IE 1.	*I got a perching stool for the kitchen and a shower stool […] if I had a shower I had to go back to bed for an hour – it absolutely wore me out and that went on for [a while]*. (Amanda, ICU)
IE 2.	*So, to pace properly you need to have a very strict timetable of rests. […] If I had a morning of doing something I would be in bed all afternoon [and it's] not a choice […] it's like ‘If I don't go to bed now, I am going to fall down’*. (Elsa, LC)
IE 3.	*You have to do things totally differently and respect your body [more, but] also find out that you can't do what you used to be able to do*. (David, ICU)
IE 4.	*[what's helped me most is] learning what my new rules are in my body […] you're building up a map of yourself and once you have the map, it becomes easier to traverse*. (Charles, LC)

**Table 3:** ‘Recovery trajectories’: Balancing hope and uncertainty.

IE 1.	*[F]or the first few days [after I came out of hospital] I continued getting stronger […] then after about 2 weeks things started to slow down […] like I was backpedaling, I was getting worse [and it's] been the same [since]*. (Kenneth, ICU)
IE 2.	*So things are improving [but when] I spoke to [my consultant] I said, ‘Oh, this recovery, it's taking so long’ he said, ‘Okay, just go back to February. […] how were you then? The same as [now]?’ […] I went, ‘Oh, no, no’. And my nurse [said], ‘You're not on the oxygen now either’. […] And I looked at them both and went, ‘Yeah okay, point made’.* (Amanda, ICU)
IE 3.	*So, I suppose every month that I can look back and think, ‘Okay, I did just a tiny bit more that month’. I feel […] good [it's] going in the right direction. The difficult thing is when you have the blips and horrible relapses […] that's really quite depressing*. (Holly, LC)
IE 4.	*I can potter around and do […] simple things […] and [the healthcare professionals] just tell me it's time [and] can take 5 years before you're totally over [admission with critical illness]*. (Ella, ICU)

**Table 4:** Illness experiences with different baselines and ‘recovery trajectories’ with distinct waymarkers.

IE 1.	*Within half an hour [of phoning for help] two paramedics were in my front room hooking me up […] giving me oxygen, checking my blood pressure. And before I knew it, I was in the back of an ambulance on the way to the hospital*. (Rod, ICU)
IE 2.	*They told me I was being taken up to intensive care, because I wasn't breathing well on my own, and I was going to be put on a [CPAP machine.] Yeah, I was scared, obviously*. (Beth, ICU)
IE 3.	*At the start [of the pandemic], Long COVID wasn't really a thing. [The media] was just showing the daily deaths, and people that were hospitalised. So […] you were kind of overlooked […] I didn't want to pester the A&E team, and my GP didn't have any answers for me either, so I was […] stuck in a hard place*. (Murray, LC)
IE 4.	*I couldn't understand why I wasn't getting better […] there I was months [after initial infection] with evolving symptoms, different systems affected, things that the news [or press conferences] hadn't spoken about […]. It was really confusing, and overwhelming, and worrying*. (Stephanie, LC)
IE 5.	*I feel guilty because […] I remember when [my mum] had chemo, her hair…she was so brave. And I think my God, I haven't had all of that [and] I feel traumatised by not having the same hair […] without a valid excuse, if that makes sense? I feel like it's less valid*. (Joanna, LC)
IE 6.	*I hadn't been to hospital, I'd never had a definitive [COVID] diagnosis and therefore I […] felt a bit of a fraud; [I would say to myself] “What's all this about? […] It's not like you've been really ill, Jane. People have been really ill.” […] I probably made assumptions about how other people were feeling about me [not recovering], which may or may not have been true*. (Jane, LC)

**Table 5:** Professional and societal responses to taking time to ‘recover’: Being granted or denied latitude by others.

IE 1.	*I came home [from hospital and] people [locally] were absolutely pouring out of their houses to talk to me and wish me well […] which gives you so much strength. It makes a huge difference […] you feel a bit like a celebrity […] which is a bit weird. A celebrity for the wrong reasons*. (Lee, ICU)
IE 2.	*I just had this huge sense of kind of hopelessness, when I came home [from hospital]. Grateful to be alive, but […] really hopeless [and] everyone wants you to be, ‘You've survived it, you did it!’ I didn't feel like that at all […] I felt like, I'd got back from war, and I didn't know how to adjust to civilian life […] [guilt is] definitely something that's stayed with me […] I dragged [my family] on a horrible journey [and] it's touched our family in a way that's changed it forever*. (Tessa, ICU)
IE 3.	*Normally people think of […] the post‐viral state, as something that you would experience for a week or two, possibly a few weeks after a very bad cold or flu. And that's something most people understand and recognise. […] so when people have spoken to me [saying], ‘Good luck with your recuperation’ […] I found myself thinking […] ‘Well actually, I'm not recuperating’ […] I'm managing […] a very different, long‐term situation [with] no indication that it's getting any better for me*. (William, LC)

## 3 Results

Radley [44, 45] argued that individual illness accounts, as shaped by and given meaning within socio‐cultural environments, can shed light on ‘worlds of illness’. He asserted that accounts of illness “do more than report the events which the person has suffered [as they are] fabricating a world of ‘illness’” [45] ^(p.779)^ and ‘articulate a person's situation in the world and, indeed, articulate that world, in which the individual will be held accountable to others’ [46] ^(p.221)^. Using these ideas, we identified the similar and different ways in which ICU and LC participants described their ‘worlds of illness’. We first detail the themes capturing similarities in their illness experiences: (i) losses, scars and unknown futures after COVID‐19; (ii) adjusting to changed selves: the hard work of striving for recovery; and (iii) ‘recovery trajectories’: balancing hope and uncertainty. Themes that demonstrate differences in the illness worlds of the two groups included: (i) illness experiences with different baselines and ‘recovery trajectories’ with distinct waymarkers; (ii) following a notional pathway vs negotiating unmapped territory; and (iii) professional and societal responses to taking time to ‘recover’: being granted or denied latitude by others.

### 3.1 Similarities in the ‘Worlds of Illness’ of ICU and LC Participants

#### 3.1.1 Losses, Scars and Unknown Futures After COVID‐19

ICU and LC participants described losses they had experienced since COVID‐19 infection and numerous ways in which their bodies, lives and selves were still changed many months, or longer, after their initial infection. Their accounts challenge the possibility of returning, physically, mentally or emotionally, to a ‘before illness’ version of themselves and, instead, suggest that their COVID experiences had permanently altered them.

ICU and LC participants highlighted bodily and physical losses, such as of strength, stamina and weight, and spoke of debilitating bodily changes and symptoms that persisted postinfection, including extreme fatigue, weakness, breathlessness, loss of taste and smell, forgetfulness and brain fog. These changes were described as resulting in a loss of normality, independence and spontaneity, leaving participants feeling they had lost their prior selves (Table 1 IEs 1‐2).

In both groups, participants reflected on the aspects of their health and life they wanted to regain, such as strength, independence and feeling like their ‘*old self*’, suggesting that recovery for some meant a return to the same state as before illness, Eddie (ICU) said he wanted help ‘*working out how to get […] back to the same level of capacity, capability that [I had] before’.* Many LC participants also spoke of their desire for a full return to their prior state but felt wary of hoping for too much (‘*To be honest, I try not to think about [whether I'll improve] because I don't know how hopeful I am’* (Philip, LC)). As COVID‐19 was a new virus when participants were interviewed, the many unknowns about their illness, and its future course, were highlighted by participants in both groups as emotionally challenging and casting doubt on the prospect of full recovery (Table 1 IE 3).

Across both groups, participants highlighted how the transformative nature of the ongoing physical and emotional effects of COVID‐19 made them unable to return to ‘normal’ as others could when the general public began to emerge out of COVID‐related lockdowns; participants felt at odds with this changing and ‘recovering’ world. While Moira (ICU) found it difficult to align the ‘*massive event*’ she had been through with the world getting back to normal *all of a sudden […] like [the pandemic] didn't happen’*, Elsa (LC) feared her lack of recovery would ‘*hit more’* and feel increasingly isolating as society shifted towards (new) ‘normal’ and she did not feel well enough to join friends in enjoying the easing of public health restrictions.

Reinfection was a significant concern for many, particularly before COVID‐19 vaccines became available. Ralph (ICU) said he was fearful of being hospitalised again: ‘*I'll be glad tomorrow when I get the initial [vaccine]’*.

#### 3.1.2 Adjusting to Changed Selves: The Hard Work of Striving for ‘Recovery’

Participants in both groups spoke of trying to adapt to their altered capacity so they could get by day‐to‐day and strive towards regaining independence. Their accounts highlighted the physical, mental and emotional toll of ‘doing’ recovery work. This included: learning how to do essential activities of daily living while managing debilitating symptoms and depleted strength and energy (e.g., using physical aids, such as shower seats, and strategies, such as pacing); rearranging their homes spatially, at least temporarily, to make them easier to navigate; and recognising and responding to signs of fatigue (Table 2 IEs 1‐2).

For both groups, much of this adjustment ‘work’ involved making sense of their changed selves and experiential learning about their diminished capacities, often after pushing themselves too far, and appropriate adaptations (Table 2 IEs 3‐4). Adjusting to changed selves also required both ICU and LC participants to rely on partners and close family members for practical and emotional support. As some ICU patients were discharged relatively quickly, due to pressures on hospitals, their care needs at home were greater than might usually be the case. Participants highlighted the necessity of this support; it was not uncommon for family members to help with activities of daily living such as showering and cooking. Adam (LC) said of his wife, ‘*I can't imagine having a carer who's better than her’*.

#### 3.1.3 ‘Recovery Trajectories’: Balancing Hope and Uncertainty

Across both groups, participants described monitoring themselves for signs of improvement or deterioration. Trajectories towards a hoped‐for recovery were heterogeneous but rarely smooth or steady in terms of their directional or temporal nature. For some, their symptoms were static, and they did not feel they were improving when interviewed (‘*I'm not in any kind of recovery phase’* (William, LC)). Others said they had improved to a degree then levelled off (‘*In the last three, 4 months I seem to have got stuck [and] I wonder is this […] as good as I'm going to get?’* (Linda, LC) or had started to regress (Table 3, IE 1).

A few ICU and LC participants suggested they were on a steady path to recovery, albeit at an extremely slow pace. Hugh (ICU) reflected on how it felt like he had ‘*to do everything in slow motion*’ when first discharged but had carefully and gradually built his ability to walk for two miles. Elsa (LC) described her progress as ‘*slow [and] fragile*’. Only one LC participant said she had fully recovered, with reservation—‘*I think I will always have a little bit of something saying, “Is this going to come back?”’* (Jane, LC). A few ICU participants also felt physically recovered when interviewed but remained afraid of reinfection.

In both groups, participants' emotions were closely linked to their understandings of the temporal and directional nature of their ‘recovery trajectory’. Times of progress were characterised by hope and relief, whereas setbacks and plateaus brought sadness, frustration and worry. The slow pace of improvement was linked to frustration or tensions between frustration and acceptance (Table 3 IEs 2‐3). Some participants were faced with coming to terms with ‘new normals’ reflecting the severity of their ongoing symptoms, either in isolation or in combination with pre‐existing or newly acquired disabilities or the effects of ageing. For example, Marzia (LC) had a chronic health condition before COVID‐19 and had experienced increased pain, worse fatigue and decreased interest in life since having Long COVID. Ella (ICU), who had severe osteoarthritis before contracting COVID‐19, suggested she needed to accept a changed version of herself and find ways of living alongside her disabilities long‐term (Table 3 IE 4).

### 3.2 Differences in the ‘Worlds of Illness’ Described by ICU and LC Participants

#### 3.2.1 Illness Experiences With Different Baselines and ‘Recovery Trajectories’ With Distinct Waymarkers

While both groups were existentially changed by COVID‐19 infection, our comparative analysis revealed marked differences in their ‘worlds of illness’. Their accounts suggested their illness experiences started from different ‘baseline’ positions and their recovery trajectories featured distinct waymarkers or interim destinations. Participants admitted to ICU early in the pandemic described the stark and dramatic baseline of their illness experience. Being admitted to ICU was a decision that was taken out of their hands by paramedics or hospital staff and could be frightening, especially because people were aware of the high mortality rates for severe COVID‐19 at the time (Table 4 IEs 1‐2).

For ICU participants, hospitalisation marked the onset of patienthood, swiftly followed by a succession of treatments and interventions beyond their control. Not only was their medical care escalated to the point of ICU admission, ICU participants also survived the harrowing and unique experience of being surrounded by others who were also critically ill with COVID‐19. Some spoke of being haunted by memories of dreams, hallucinations and events they experienced or witnessed in ICU, such as the illness and deaths of others with COVID‐19. Some suggested this was ‘*like mental torture*’ (Lee, ICU) and psychologically scarring (‘*COVID is not a thing that comes and goes. It's always with you, somehow, in your mind, it's always there’* [Rayhanna, ICU]).

By contrast, LC participants highlighted the comparative invisibility of their illness experience ‘baseline’, which took place behind closed doors, was not recognised or broadcast by mainstream media, and was unfamiliar to the medical profession (Table 4 IE 3).

For many LC participants, their early days, weeks and months of illness were characterised by feeling their lives had been ‘*turned upside down*’ (Fraser, LC) and an inability to explain their ongoing symptoms. Many, including some who were healthcare professionals, were bewildered by their symptoms. For example, Stephanie (Table 4 IE 4) said the absence of media attention on persistent symptoms compounded her concerns. It was perhaps the stark contrast between how visible, closely monitored and reported hospitalisations for severe COVID‐19 were in the media and how invisible the experience of Long COVID was in the early stages of the pandemic which led some LC participants to compare themselves to people who had been critically ill or had died as a result of COVID‐19, and indeed other illnesses. While hospital, and especially ICU, admission confirmed legitimate patient status for those critically ill with COVID‐19, participants with LC suggested their struggle for legitimacy gave rise to feelings of guilt for struggling with their symptoms, especially when testing for COVID‐19 infection was not available (Table 4 IEs 5‐6)

Signs of improvement in the ‘recovery trajectories’ described also differed. For ICU participants, key markers of progress were being moved out of ICU and being discharged from hospital. Some recalled staff clapping them out of ICU to celebrate this major milestone. Lee (ICU) recalled the significance of hospital discharge as a waymarker on his illness trajectory; it represented being ‘*on the mend*’.

For LC participants, ‘recovery trajectories’ featured few concrete markers of progress comparable to hospital discharge, although a few described more nebulous signs of improvement, including: ‘*fewer days of having to sleep all the time*’ (Gemma); ‘*having extra energy*’ at the end of the day (Elsa); and beginning to live more spontaneously (‘*to be most of the day out of the house without having to be too rigid in my planning*’ [Elliott]).

#### 3.2.2 Following a Notional Pathway vs Negotiating Unmapped Territory

Participants in both groups faced the task of making sense of and adjusting to their changed bodies. Nevertheless, ICU and LC participants' accounts differed in the extent to which their sense‐making work and practices of patienthood were recognised and supported. For some (by no means all) ICU participants this was underpinned by explanations for losses they had experienced, often informed by ICU staff (through conversations, diaries or medical notes) or family members. This information helped them understand how critically ill they had been and the experiences from which their body needed to recover. For example, Rod said ‘*I got told in intensive care I lost muscle mass in my legs, so my legs felt like jelly […] because I was laid not moving for 11 days’*. Some ICU participants described how, following discharge, they had ongoing professional support to inform their expectations of recovery, guide them in adapting to their changed capacity and advise on strategies for regaining strength. However, standard systems of care were stretched due to the pandemic and access to ongoing support varied greatly. While Lee said he felt ‘*there were a lot of people from the NHS that were concerned […] making sure that I was doing everything correctly [after discharge]*’, Eddie said professional support dropped off significantly following discharge, leaving him ‘*working out how to get better*’ without a rehabilitative pathway. Thus, although an infrastructure existed for post‐ICU care generally, which could serve as a notional pathway for ICU patients, healthcare professionals caring for COVID‐19 ICU patients did not seem to have specific protocols for caring for patients with this new virus nor resources to provide ongoing care for so many patients.

The LC participants did not have the benefit of medical recognition or explanations for their symptoms, nor was there any notional healthcare pathway to follow. William expressed his frustration: ‘*it would be very nice to know why it is that my body feels totally exhausted just doing [gentle exercise, when] I used to climb mountains’.* LC participants often reported feeling unsupported by healthcare professionals in their efforts to make sense of and adjust to their changed selves. Participants were aware that the lack of consistent and clear guidance from healthcare professionals was because there was little evidence about treatments nor experience of advising patients how to manage symptoms, especially early in the pandemic. As a result, LC participants developed alternative practices to manage their symptoms. Gemma, for example, reported being told by healthcare professionals ‘*to push through*’ her fatigue by increasing physical activity levels daily, but later decided, when this approach left her “*floored*”, that she “*was going to ignore that advice and just pace*” herself. Some reported seeking help from private healthcare practitioners who some described as helpful and supportive. However, for many it was a case of trying to work out for themselves, or through other LC patients in online support groups, the best ways of adjusting to symptoms.

#### 3.2.3 Professional and Societal Responses to Taking Time to ‘Recover’: Being Granted or Denied Latitude by Others

The ‘worlds of illness’ which ICU and LC participants inhabited also differed in relation to others' responses to their ‘recovery’. ICU participants (particularly those admitted early in the pandemic) described receiving considerable support and strength from the help and good wishes from ICU/hospital staff, friends, other ICU survivors, and people, even strangers, in their local community (Table 5 IE 1).

Their accounts sometimes suggested they were ascribed hero status by people in their wider social networks. Indeed, Martin, a member of a support group for mechanically ventilated COVID‐19 patients, had recovered to such an extent that he had managed a sustained return to full‐time employment and described feeling touched when called ‘*an inspiration*’ by group members. However, not all ICU participants felt easy about being ascribed a hero or survivor identity. In describing her struggles with survivor guilt and concerns about the trauma her family had sustained, Tessa described a strong disconnect between her feelings and her perceptions of others' expectations of how she should feel (Table 5 IE 2).

While ICU participants were recognised as ‘legitimate’ patients and their survival celebrated by those around them, LC participants' accounts suggested others did not recognise their need to (take time to) recover, suggesting their patienthood was not ascribed comparable authenticity. Instead, they reported feeling that others assumed they were already on a pathway to recovery. This particularly applied to people outside immediate family who were unlikely to see them on “*bad days*” when most debilitated by their symptoms (Table 5 IE 3). LC participants' accounts highlighted how people around them were confused by their ongoing symptoms and how to respond to the fact they had not yet returned to their prior state of health. Early in the pandemic, lockdown restrictions meant that the ‘work’ LC participants did to adjust to their changed selves was not visible to wider social networks, being only witnessed by partners, immediate family and carers. This lack of visibility may have contributed to the surprise often expressed by others, and certainly people they saw infrequently, on hearing or seeing that they were still experiencing symptoms. Additionally, LC participants described ways in which people, perhaps as part of their own sense‐making work, proffered alternative explanations for ongoing symptoms. Stephanie said that people's responses suggested they were ‘*subconsciously, perhaps, attributing a lot of what I've been going through to psychiatric symptoms, which isn't right*’ and that even people who were close to her had tried ‘*to reason through*’ her illness by ‘*attributing the symptoms to something else’, “Well, could you be menopausal?’ […] ‘Is it your thyroid?’ […] they just want something concrete. […] I have found that really difficult*.” Although LC participants could see people around them trying to make sense of their symptoms and attribute them to illnesses with better understood trajectories, this was experienced as emotionally challenging and appeared to cast doubt on their status as legitimate patients.

## 4 Discussion

This paper aims to contribute to a wider understanding of what ‘recovery’ means, by comparing accounts from adults who were admitted to ICU with COVID‐19 and those with Long COVID in the early stages of the pandemic.

Underpinning all participants' narratives, regardless of patient group, was the novel and unknown nature of COVID‐19 and scant healthcare knowledge and services available at the time they were interviewed. As we [23] and others [33, 36, 39] have found in relation to the multiple and intersecting unknowns associated with a new emerging disease, “the uncertain future is a constant presence” [11]. For participants in the current study, this shaped their accounts and arose from there being no tried and tested ‘road map’ to guide either group towards regaining health, or to a clear point of completion of that quest. Even for ICU participants, pathways to regaining health could be notional at best.

Our comparative analyses suggest the ‘worlds of illness’ of ICU and LC participants were similar in some respects. Accounts from both groups complicate idealised and widely‐held notions of recovery as a linear transition between binary states of illness and wellness [36, 39, 47]. Similar to others' descriptions of the ‘end of normality’ [3] ^(p.1755)^ inflicted by Long COVID, participants in both groups described how their body and sense of self were altered, sometimes profoundly, by the physical, mental and emotional losses and scars resulting from COVID‐19 infection [22, 48], and (for the ICU group) life‐saving treatment. Participants in both groups questioned the achievability of full recovery, in the sense of returning to a ‘before illness’ version of themselves, yet did not know what to expect of or hope for their future selves either [11, 36].

We describe elsewhere the ‘adjustment work’ done by people with Long COVID in relation to the challenges faced on attempting to return to employment [32]. In the current study, we highlight how adjusting to their changed selves required participants to undertake hard physical and emotional work, such as striving to make sense of their symptoms, discovering and respecting the ‘*new rules*’ of their body, often through trial and error [49] or ‘embodied experimentation’ [38], as others have described. We identified heterogeneity in the ‘recovery trajectories’ described by both groups, as identified for people with Long COVID [39, 47]. Positive emotions characterised times of ‘progress’ whereas slowing improvement or regressions engendered sadness, frustration and concerns about the future. Participants' feelings may be founded on their own expectations, and perhaps their perceptions of others’, that ‘successful recovery’ comprises steady (even rapid) trajectories towards improvement, suggesting the active influence of a ‘recovery imperative’ which casts certain types of recovery as more desirable and acceptable than others [20, 21].

Key differences in ‘worlds of illness’ stemmed from the fact that ICU participants' hospitalisation conferred them status as legitimate patients in ‘medically clear and understandable ways’ [14] ^(p.10)^ whereas LC participants' ‘patienthood’ was neither evidenced nor authenticated in the same way. ICU participants recounted piecing together memories of being surrounded by others with the same unknown virus and witnessing severe illness and death. Thus their trajectories started from a stark baseline, although blurred by uncertainty as to what was real; their very survival symbolised them reaching a major waymarker on their ‘recovery map’. In contrast, LC participants' illness baseline happened behind the closed doors of their homes, characterised by a chasm between their symptom experiences and lack of medical knowledge or a diagnosis [14]. This challenged their claim to a patient status or ‘survivor identity’ [7], in contrast to ICU participants. Indeed, initially, LC participants could only compare their experiences of COVID‐19 with mainstream media images of severe disease and death in ICU [33], perhaps contributing to some participants feeling they should have comparatively little to complain about. While (at least early in the pandemic) ICU participants' trajectories were dotted with interim destinations (e.g., moving out of ICU, being discharged from hospital) this was not the case for LC participants whom we have previously described as ‘vanguard patients’ [27]; for these people there was not even a notional ‘map’ of how they might return to health, and, as others have also reported, it was unclear whether they ever could [3, 11, 36].

The ‘worlds of illness’ which ICU and LC participants navigated were further differentiated by professional and societal responses they encountered. Indeed, it was with regard to the meanings and social interactions associated with their ‘recovery’ that the differences between the socio‐cultural worlds described by the two groups were especially salient. Whether they were met with admiration and sympathy or suspicion and frustration, our findings demonstrate that both forms of societal response can be challenging to navigate. Many ICU participants said they were told they would need time to recover, even if an overwhelmed health service was too stretched to provide sufficient support [26]. Many we interviewed were buoyed by support and encouragement from partners, families and wider social networks, who, in acknowledging the symbolic significance of their hospitalisation, recognised their patienthood and ascribed them a “reframed ‘survivor identity’” [7]. ^(p.86)^ These responses allowed grace for recovery to be fragile, ongoing and incomplete, whilst celebrating ICU patients as inspirational for elements of ‘successful’ recovery, defined for example by a return to paid employment. Those with LC, by contrast, spoke of a lack of recognition and clear guidance from healthcare professionals regarding how to adjust to and treat their symptoms, requiring them to carry out sense‐making work in isolation or, in time, alongside others with LC online [50]. Cheston et al. [11] highlight the ‘social suffering’ experienced by people with LC, stemming from a ‘twofold lack of support—medical and social’. In the current study, instead of being granted latitude to be ill and time to recover, LC participants described surprised or confused societal responses to their ongoing symptoms. Indeed, the contested nature of LC was brought into sharp relief as participants recounted others' efforts to explain their ongoing symptoms by reaching for what they perhaps believed to be ‘real’, more likely, or more credible causes [21, 36]. The differing social responses perhaps reflect the meanings ascribed by others to the illness experiences of our two groups; while ICU admission confers legitimate patienthood, LC represents a ‘messy category’ [14] ^(p.15)^ by comparison wherein patienthood is contested and questioned.

A strength of this study lies in the robust and significant corpus of data (93 in‐depth interviews). Participants also gave permission for interview extracts, redacted according to their wishes, to be used for purposes including teaching, secondary analysis and on an online platform (hexi.ox.ac.uk). Although all participants had the option of safeguarding their identity by making their data fully anonymous, we acknowledge this may have led some people to censor aspects of their experiences. A further limitation is the limited ability to explore longer term perspectives of recovery. Our data were not longitudinal, and interviews were conducted between February 2021 and July 2022. Hence these ‘recovery narratives’ are somewhat truncated and must be understood as situated within early stages of the pandemic when the recency of COVID‐19 meant healthcare knowledge and treatment pathways were nascent. Longitudinal research with a variety of COVID‐19 patient groups could elucidate longer‐term ‘recovery’ trajectories [34, 36]. Indeed, it is important to follow lived experiences of ongoing symptoms of COVID‐19 to assess whether people's abilities to reconcile illness and identity will be affected by improvements in understandings of Long COVID in the future. The high numbers of people affected by Long COVID may in time challenge wider notions of recovery [36].

## 5 Conclusions

We have presented findings of comparative analyses which illustrate how the ‘worlds of illness’ described by these two participant groups converged and diverged in illuminating ways, showing how notions of recovery and ways of striving towards it are impacted by similarities and differences in illness experiences, and others' reactions to these. While the paper draws on experiences of COVID‐19, the findings have implications for wider understanding of recovery, which has received little attention in qualitative social science. Similarities across the participant groups' accounts related to daily symptom experiences and the functional ways they strived towards the accomplishment and recovery of health. Differences related to social accomplishments of patienthood and how these were recognised or disputed by others. Of note were the differing professional and societal responses reported by the two groups, which lend insight into how the social processes of recovery are entwined with (wider) understandings of the illness.

Our findings have clinical implications. They emphasise the importance of ensuring people are made to feel their illness experiences are legitimate, regardless of hospitalisation status, formal diagnosis or lack of medical knowledge and pathways. In relation to healthcare practices, this involves listening carefully to patients as individuals and demonstrating belief of their experiences. Our findings also highlight the value of emphasising to patients and family members the different permutations and lack of linearity, that recovery can take, to help them guard against internalising idealised notions of recovery which can engender negative experiences of a perceived lack of progress. When illness severely disrupts everyday life, profoundly altering people's sense of self, the desire to return to a ‘before illness’ version of the self is entirely understandable, both for those experiencing illness and those caring for/about them. However, if accepted societal understandings of recovery are limited, prescriptive and experienced as imperative, there is a danger that illness trajectories which are faltering, unexpectedly slow or not moving towards a person's prior state of health will be imbued with ‘failure’. Acknowledging and valuing alternatives to the ‘recovery imperative’, within healthcare relationships and wider societal understandings, is a vital part of improving illness experiences, which, paradoxically, may facilitate a more positive use of (limited) energy.

## Author Contributions

**Alice MacLean:** substantial contribution to conception of design and to acquisition of data. Leading role in analysis and interpretation, wrote all drafts of paper. **Annelieke Driessen:** substantial contribution to conception of design and to acquisition of data. Leading role in analysis and interpretation, leading role in critical review of all drafts of paper. **Lisa Hinton:** substantial contribution to conception of design, interpretation of data, critical review of earlier drafts of paper. **Sarah Nettleton:** substantial contribution to conception of design, interpretation of data, leading role in critical review of earlier drafts of paper. **Cervantee Wild:** substantial contribution to conception of design and to acquisition of data. Contribution to analysis and interpretation, critical review of drafts of paper. **Eilidh Anderson:** substantial contribution to analysis and interpretation, contribution to first draft of paper and critical review of later drafts of paper. **Ashely Brown:** substantial contribution to conception of design and to acquisition of data. Contribution to analysis and interpretation, critical review of drafts of paper. **Pat Hoddinott:** contribution to analysis and interpretation, critical review of drafts of paper. **Calum O'Dwyer:** contribution to analysis and interpretation, critical review of drafts of paper. **Sue Ziebland:** contribution to conception of design, analysis and interpretation, critical review of drafts of paper. **Kate Hunt:** substantial contribution to conception of design and to acquisition of data, analysis and interpretation, critical review of earlier drafts of paper.

## Ethics Statement

Our study was approved by the Berkshire Ethics Committee (12/SC/0495). All participants provided recorded verbal informed consent which was obtained at the start of the interview by the researcher conducting it.

## Conflicts of Interest

The authors declare no conflicts of interest.

## Data Availability

Data are available on reasonable request. The University of Oxford holds the copyright for the full interview transcripts and may grant data sharing permission on request.

## References

[1] Coronavirus: Action Plan (UK Government, 2020), accessed May 01, 2024, https://assets.publishing.service.gov.uk/media/5e5e2e91e90e071110454391/Coronavirus_action_plan_-_a_guide_to_what_you_can_expect_across_the_UK.pdf.

[2] F. Callard . Very, Very Mild: Covid‐19 Symptoms and illness Classification, accessed May 14, 2024, https://somatosphere.com/2020/mild-covid.html/.

[3] J. Ireson , A. Taylor , E. Richardson , B. Greenfield , and G. Jones , “Exploring Invisibility and Epistemic Injustice in Long Covid‐A Citizen Science Qualitative Analysis of Patient Stories From an Online Covid Community,” Health Expectations: An International Journal of Public Participation in Health Care and Health Policy 25, no. 4 (August 2022): 1753–1765, 10.1111/hex.13518. 35557480 PMC9327841

[4] M. Fricker , Epistemic Injustice: Power and the Ethics of Knowing (Oxford University Press, 2007).

[5] Y. Friedman , “On Recovery: Re‐Directing the Concept by Differentiation of Its Meanings,” Medicine, Health Care, and Philosophy 24, no. 3 (2021): 389–399, 10.1007/s11019-021-10014-7. 33811591 PMC8019304

[6] T. Mladenov and I. Dimitrova , “Epistemic Injustice as a Bridge Between Medical Sociology and Disability Studies,” Sociology of Health & Illness 45, no. 6 (July 2023): 1146–1163, 10.1111/1467-9566.13479. 35543112

[7] A. C. Gonçalves , A. Williams , C. Koulouglioti , et al., “Surviving Severe COVID‐19: Interviews With Patients, Informal Carers and Health Professionals,” Nursing in Critical Care 28, no. 1 (January 2023): 80–88, 10.1111/nicc.12779. 35561020 PMC9348004

[8] Herridge Ms , Azoulay E. Outcomes After Critical Illness,” Reply New England Journal of Medicine 389, no. 4 (July 2023): 382–383, 10.1056/NEJMc2304264. 37494501

[9] J. Dumit , “Illnesses You Have to Fight to Get: Facts as Forces in Uncertain, Emergent Illnesses,” Social Science & Medicine 62, no. 3 (February 2006): 577–590, 10.1016/j.socscimed.2005.06.018. 16085344

[10] H. E. Davis , L. McCorkell , J. M. Vogel , and E. J. Topol , “Long Covid: Major Findings, Mechanisms and Recommendations,” Nature Reviews Microbiology 21 (2023): 133–146, 10.1038/s41579-022-00846-2. 36639608 PMC9839201

[11] K. Cheston , M. L. Cenedese , and A. Woods , “The Long or the Post of It? Temporality, Suffering, and Uncertainty in Narratives Following Covid‐19,” Journal of Medical Humanities 46 (November 2023): 3–20, 10.1007/s10912-023-09824-y. 37962719 PMC11805856

[12] E. Ladds , A. Rushforth , S. Wieringa , et al., “Persistent Symptoms After Covid‐19: Qualitative Study of 114 “Long Covid” Patients and Draft Quality Principles for Services,” BMC Health Services Research 20, no. 1 (December 2020): 1144, 10.1186/s12913-020-06001-y. 33342437 PMC7750006

[13] B. Oronsky , C. Larson , T. C. Hammond , et al., “A Review of Persistent Post‐Covid Syndrome (PPCS),” Clinical Reviews in Allergy & Immunology 64, no. 1 (February 2023): 66–74, 10.1007/s12016-021-08848-3. 33609255 PMC7896544

[14] C. Moretti and K. K. Barker , “Suffering Without Remedy: The Medically Unexplained Symptoms of Fibromyalgia Syndrome and Long Covid,” Social Sciences 13, no. 9 (2024): 450.

[15] Office for National Statistics (ONS). Self‐Reported Coronavirus (COVID‐19) Infections and Associated Symptoms, England and Scotland: November 2023 to March 2024, accessed February 13, 2025, https://www.ons.gov.uk/peoplepopulationandcommunity/healthandsocialcare/conditionsanddiseases/articles/selfreportedcoronaviruscovid19infectionsandassociatedsymptomsenglandandscotland/november2023tomarch2024.

[16] A. Cheshire , D. Ridge , L. V. Clark , and P. D. White , “Sick of the Sick Role: Narratives of What “Recovery” Means to People With Cfs/ME,” Qualitative Health Research 31, no. 2 (2021): 298–308, 10.1177/1049732320969395. 33176575 PMC7750673

[17] A. Woods , A. Hart , and H. Spandler , “The Recovery Narrative: Politics and Possibilities of a Genre,” Culture, Medicine and Psychiatry 46, no. 2 (June 2022): 221–247, 10.1007/s11013-019-09623-y. 30895516 PMC9034998

[18] A. W. Frank , The Wounded Storyteller. Body, Illness, and Ethics (University of Chicago Press, 1997).

[19] T. Parsons , The Social System (The Free Press, 1951).

[20] W. O'Brien , “The Recovery Imperative: A Critical Examination of Mid‐Life Women's Recovery From Depression,” Social Science & Medicine 75, no. 3 (2012): 573–580. 22580074 10.1016/j.socscimed.2012.03.034

[21] D. Karadzhov , “Personal Recovery and Socio‐Structural Disadvantage: A Critical Conceptual Review,” Health: An Interdisciplinary Journal for the Social Study of Health, Illness and Medicine 27, no. 2 (March 2023): 201–225, 10.1177/13634593211014250. PMC9923205 33962518

[22] R. Collins , F. Vallières , and G. McDermott , “The Experiences of Post‐ICU COVID‐19 Survivors: An Existential Perspective Using Interpretative Phenomenological Analysis,” Qualitative Health Research 33, no. 7 (2023): 589–600, 10.1177/10497323231164556. 37023365 PMC10083706

[23] A. Driessen , A. Navarro de Souza , M. E. P. Castellanos , M. V. Tuma de Oliveira , E. L. Carvalho , and L. Hinton , “Navigating Uncertainties In Critical Care With Covid‐19: A Cross Country Analysis of Patient Narratives From Brazil and the United Kingdom,” SSM ‐ Qualitative Research in Health 5 (2024): 100363, 10.1016/j.ssmqr.2023.100363.

[24] R. Norouzadeh , M. Abbasinia , Z. Tayebi , et al., “Experiences of Patients With COVID‐19 Admitted to the Intensive Care Units: A Qualitative Study,” Journal of Patient Experience 8 (2021): 23743735211007359, 10.1177/23743735211007359. 34179418 PMC8205342

[25] M. Onrust , A. Visser , N. van Veenendaal , et al., “Physical, Social, Mental and Spiritual Functioning of COVID‐19 Intensive Care Unit‐Survivors and Their Family Members One Year After Intensive Care Unit‐Discharge: A Prospective Cohort Study,” Intensive & Critical Care Nursing 75 (2023): 103366, 10.1016/j.iccn.2022.103366. 36528460 PMC9726690

[26] J. Ganton , A. Hubbard , and K. Kovacs Burns , “Patients With COVID‐19 Share Their Experiences of Recovering at Home Following Hospital Care Transitions and Discharge Preparation,” Health Expectations: An International Journal of Public Participation in Health Care and Health Policy 25, no. 6 (2022): 2862–2875, 10.1111/hex.13595. 36134451 PMC9538741

[27] A. MacLean , K. Hunt , A. Brown , et al., “Negotiation of Collective and Individual Candidacy for Long Covid Healthcare In the Early Phases of the Covid‐19 Pandemic: Validated, Diverted and Rejected Candidacy,” SSM Qualitative Research in Health 3 (2023): 100207, 10.1016/j.ssmqr.2022.100207. 36507117 PMC9721377

[28] F. Verdonk , D. Feyaerts , R. Badenes , et al., “Upcoming and Urgent Challenges in Critical Care Research Based on COVID‐19 Pandemic Experience,” Anaesthesia, Critical Care & Pain Medicine 41, no. 5 (October 2022): 101121, 10.1016/j.accpm.2022.101121. PMC9245393 35781076

[29] H. Humphreys , L. Kilby , N. Kudiersky , and R. Copeland , “Long Covid and the Role of Physical Activity: A Qualitative Study,” BMJ Open 11, no. 3 (March 2021): e047632, 10.1136/bmjopen-2020-047632. PMC7948149 33692189

[30] J. Shelley , J. Hudson , K. A. Mackintosh , et al., “‘I Live a Kind of Shadow Life’: Individual Experiences of COVID‐19 Recovery and the Impact on Physical Activity Levels,” International Journal of Environmental Research and Public Health 18, no. 21 (October 2021): 11417, 10.3390/ijerph182111417. 34769934 PMC8583577

[31] F. Lowenstein I Rested My Way to Recovery From Long Covid. I Urge Others to Do the Same. The Guardian. 21/06/2021, accessed May 14, 2024, https://www.theguardian.com/commentisfree/2021/jun/21/long-covid-recovery-coronavirus.

[32] E. Anderson , K. Hunt , C. Wild , S. Nettleton , S. Ziebland , and A. MacLean , “Episodic Disability and Adjustments for Work: The ‘Rehabilitative Work’ of Returning to Employment With Long Covid,” Disability & Society (2024): 1–23, 10.1080/09687599.2024.2331722.

[33] K. Nielsen and J. Yarker , “It's a Rollercoaster”: The Recovery and Return to Work Experiences of Workers With Long Covid,” Work & Stress 38, no. 2 (2024): 202–230, 10.1080/02678373.2023.2286654.

[34] Z. Al‐Aly and E. Topol , “Solving the Puzzle of Long Covid,” Science (New York, N.Y.) 383, no. 6685 (February 2024): 830–832, 10.1126/science.adl0867. 38386747

[35] C. J. Atchison , B. Davies , E. Cooper , et al., “Long‐Term Health Impacts of COVID‐19 Among 242,712 Adults in England,” Nature Communications 14, no. 1 (2023): 6588, 10.1038/s41467-023-41879-2. PMC10598213 37875536

[36] N. J. Spence , D. Russell , E. D. Bouldin , C. M. Tumminello , and T. Schwartz , “Getting Back to Normal? Identity and Role Disruptions Among Adults With Long Covid,” Sociology of Health & Illness 45, no. 4 (May 2023): 914–934, 10.1111/1467-9566.13628. 36880317

[37] C. Fang , S. A. Baz , L. Sheard , and J. D. Carpentieri , “‘I Am Just a Shadow of Who I Used to Be’‐Exploring Existential Loss of Identity Among People Living With Chronic Conditions of Long Covid,” Sociology of Health & Illness 46, no. 1 (2024): 59–77, 10.1111/1467-9566.13690. 37391994

[38] M. Harrison , T. Rhodes , and K. Lancaster , “Constitution of Long Covid Illness, Patienthood and Recovery: A Critical Synthesis of Qualitative Studies,” BMJ Open 14, no. 3 (March 2024): e083340, 10.1136/bmjopen-2023-083340. PMC10982801 38548364

[39] R. L. Knight , K. A. Mackintosh , J. Hudson , J. Shelley , Z. L. Saynor , and M. A. McNarry , “Battling the Unknown: Using Composite Vignettes to Portray Lived Experiences of COVID‐19 and Long‐Covid,” PLoS One 18, no. 4 (2023): e0284710, 10.1371/journal.pone.0284710. 37099534 PMC10132598

[40] I. T. Coyne , “Sampling In Qualitative Research. Purposeful and Theoretical Sampling; Merging or Clear Boundaries?,” Journal of Advanced Nursing 26, no. 3 (1997): 623–630, 10.1046/j.1365-2648.1997.t01-25-00999.x. 9378886

[41] R. Sokol , E. Fisher , and J. Hill . “Identifying Those Whom Health Promotion Hardly Reaches: A Systematic Review,” Evaluation & the Health Professions 38, no. 4 (2015): 518–537, 10.1177/0163278715605883. 26405265

[42] C. Pope , S. Ziebland , and N. Mays , “Analysing Qualitative Data.” in Qualitative Research in Health Care, ed. N. Mays . (Wiley‐Blackwell, 2020). Fourth Edition ed.

[43] S. Ziebland and A. McPherson , “Making Sense of Qualitative Data Analysis: An Introduction With Illustrations From DIPEx (Personal Experiences of Health and Illness),” Medical Education 40, no. 5 (May 2006): 405–414, 10.1111/j.1365-2929.2006.02467.x. 16635119

[44] A. Radley , Worlds of Illness. Biographical and Cultural Perspectives on Health and Disease (Routledge, 1993).

[45] A. Radley , “The Aesthetics of Illness: Narrative, Horror and the Sublime,” Sociology of Health & Illness 21, no. 6 (1999): 778–796.

[46] A. Radley and M. Billig , “Accounts of Health and Illness: Dilemmas and Representations,” Sociology of Health & Illness 18, no. 2 (1996): 220–240.

[47] B. Figueiredo , J. Sheahan , S. Luo , et al., “Journey Mapping Long Covid: Agency and Social Support for Long‐Hauling,” Social Science & Medicine (1982) 340 (2024): 116485, 10.1016/j.socscimed.2023.116485. 38056307

[48] C. M. Thompson , S. Babu , E. Gerlikovski , et al, “Living with Long Covid: A Longitudinal Interview Study of Individuals' Communicative Resilience Through the “Long Haul,” Health Communication 39, no. 10 (2023): 2135–2151, 10.1080/10410236.2023.2257941. 37733066

[49] P. Page , A. Simpson , and L. Reynolds , “Constructing a Grounded Theory of Critical Illness Survivorship: The Dualistic Worlds of Survivors and Family Members,” Journal of Clinical Nursing 28, no. 3–4 (February 2019): 603–614, 10.1111/jocn.14655. 30182420

[50] F. Callard and E. Perego , “How and Why Patients Made Long Covid,” Social Science & Medicine (1982) 268 (January 2021): 113426, 10.1016/j.socscimed.2020.113426. 33199035 PMC7539940

